# Pseudotumoral Hydatid Cyst: Report of a Case

**DOI:** 10.1155/2009/137956

**Published:** 2009-08-23

**Authors:** Ioannis E. Petrakis, Evaggelia Grysbolaki, Stefanos Paraskakis, Theodore Lagoudis, Demetrios Filis, George Chalkiadakis

**Affiliations:** Department of General Surgery, University Hospital of Heraklion, Medical School of Crete, 114 Akadimias Avenue, Heraklion, 71305 Crete, Greece

## Abstract

Hydatidosis due to *Echinococcus granulosus* is an endemic parasitic zoonosis characterized by worldwide distribution particularly in Mediterranean countries. The most commonly involved anatomical locations are the liver and lung. Occasionally the cyst may progressively increase in size, mimicking gross ascites or intrabdominal tumor. Herein, are reported a case of a 40-year-old patient with a giant exophytically expanded hepatic echinococcus cyst, misdiagnosed as an abdominal malignancy during formal investigation. The patient was admitted to the hospital complaining for mild diffuse abdominal tenderness, moderate abdominal pain, nausea, diarrhoea, and vomiting. A CT scan revealed the presence of a giant abdominal mass 25 × 21 × 14 cm, resembling a tumor, adherent to the liver edges and parietal peritoneum, displacing intestinal loops. During the ensuing days the patient's clinical condition worsened, and he became febrile. Exploratory laparotomy was performed, and an exophytically grown giant liver hydatid cyst was removed, despite the radiological findings and the preoperative clinical suspicion.

## 1. Introduction

Hydatidosis due to *Echinoccocus granulosus* is an endemic parasitic zoonosis characterized by worldwide distribution with prevalence in the Middle East and the Mediterranean countries [1]. Liver is one of the most frequently involved organs. Liver hydatid cysts are characterized by insidious development in the majority of patients, with the potential for peritoneal dissemination of daughter hydatid cysts. Occasionally the cyst may progressively increase in size, mimicking gross ascites or liver tumor. Often the large size of the cyst makes the diagnosis extremely difficult [1, 2]. However, the aim of this case report is to present a rare case of a giant hydatid cyst grown exophytically from the right lobe of the liver resembling the radiological findings of a tumoral mass.

## 2. Case Report

A 40-year-old man was admitted to our hospital with pain in the right upper quadrant and epigastric region beginning 20 days before, after weightlifting and loss of appetite associated with nausea. The patient had no history of liver disease, jaundice, or changing in bowel habits. Fever appeared the last day before the admission probably because of partial rupture of the cyst as a result of abrupt increase in intraabdominal pressure and spillage of the content into the peritoneal cavity.

**Figure 1:**
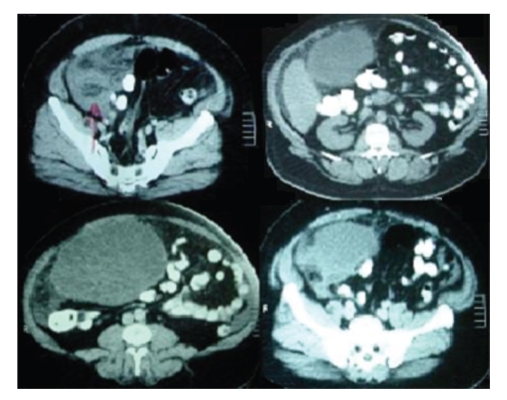
Abdominal CT scan showing a huge heterogeneous cystic mass occupying the entire right side of the abdomen and extending down to the pelvis.

**Figure 2:**
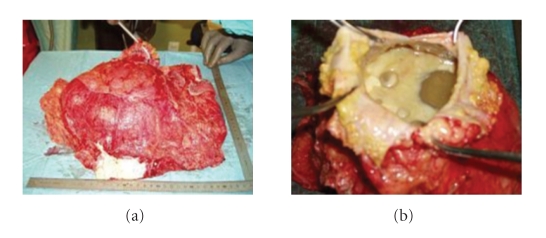
Hydatid cyst and pericystectomy material after surgery (a), open cyst (b).

Abdominal examination revealed right flank tenderness and abdominal enlargement with dullness on percussion without shifting dullness indicating either fluid collection or mass. Further diagnostic evaluation raised the clinical suspicion of abdominal malignancy. On the abdominal ultrasonography, there was a large >10 cm, solid, hyperechogenic, cystic lesion with heterogeneous pseudotumor appearance and presence of large amount of perihepatic, intraperitoneal, and pelvic ascitic fluid nonpathognomonic of hydatid disease. The gallbladder was normal, and no *other* lesions were seen. Results of laboratory tests except of a mild leucocytosis otherwise were normal. Abdominal computed tomography (CT scan) revealed the presence of a 25 cm × 21 cm × 14 cm cystic mass strongly adherent to the right hepatic lobe. The cyst filled the entire right and median side of the abdominal cavity, displacing the intestinal loops to the left, and was extended down into the pelvis. The patient was taken into operation with the diagnosis of a large cystic with solid features tumoral mass (Figure 1). Upon direct examination, a huge thin-walled cyst, which grew exophytically from the anterior part of the right hepatic lobe containing daughter vesiculae, was found to occupy the entire right side of the abdomen. The pericyst was found strictly adherent to the parietal peritoneum and the bowel loops. Yellow, nonprulent gelatinous matrix with daughter cysts at various stages of degeneration was found in the cyst. The cultures that were obtained intraoperatively from this fluid were negative for bacterial infection. Microscopy of the cystic fluid showed hooklets of *E. granulosus* (Figure 2).

The patient made an uneventful recovery and was discharged the 8th postoperative day on albendazole (400 mg twice daily) for two months. There was not recurrence of hydatid cyst at nine months follow up of the patient.

## 3. Discussion

Differential diagnosis of a hydatid cyst, from other abdominal cystic lesions, is sometimes difficult. The challenge in this case laid in its clinical presentation. The clinical presentation of hydatid disease is largely asymptomatic until complications occur [1]. When symptoms appear, pain is the commonest symptom of hydatid disease. Fever with chills and rigors can occur if the cyst is secondarily infected. Rupture of the cyst is the most common complication and can be the result of trauma or pressure from the growing cyst and may occur into the biliary duct, thoracic cavity, adjacent hollow viscera, or peritoneal cavity [2]. This may result in anaphylactic shock and formation of localized or generalized secondary echinococcosis.

Structurally, a hydatid cyst is composed by an innermost cellular membrane, the germinal layer, or endocyst which gives rise to scolices and daughter cysts and an external acellular cuticule, the ectocyst or laminated membrane, which is secreted by the parasite. In our case the ectocyst was adherent strictly to the parietal peritoneum, and meticulous dissection was needed. Daughter cysts can often simulate the appearance of cystic tumors [3] and can be easily misdiagnosed as in our case, however, rarely appear in large dimension without previous symptoms. Within the cyst fluid can be found “hydatid sand,” made up of sloughed capsules and scolices. Cyst is surrounded by a tough elastic capsule on pericyst formed by the host. According to World Health Organization-Informal Working Group on Echinococcosis (WHO-IWGE) classification based on sonographic analysis of the morphology and structure of the hydatid cysts five categories are recognized [4]. Types CE2 and CE3 represent the typical hydatid cysts; types CE1 and CE5 are suggestive of hydatid cysts; type CE4, simulates a cystic tumor. 

Echinococcal cysts are mostly found in the liver (60%–70% of cases), lungs (10%–25%), and less frequently involved anatomical locations such as brain [5], bones [6], and heart [7]. Giant hydatid cysts are extremely rare. Uncomplicated liver cysts are always asymptomatic initially, and it remains so for longer periods, especially when only small, well-encapsulated, or calcified cysts are present. The cyst herein reported is an extremely rare large asymptomatic cyst, largely than CE4 type, well-encapsulated, and because of the size, strongly adherent to the parietal peritoneum but not calcified, presenting with the characteristics of a giant intrabdominal cystic tumor. Abdominal pain, hepatomegaly, or a palpable mass in the right upper quadrant are the most common clinical presentations for patients with liver echinococcosis. Early diagnosis is life saving as potentially lethal complications such as anaphylactic shock due to perforation of the cyst may occur. Complete elimination of the parasite from the organism and prevention of recurrence of disease constitute the ideal treatment for hydatid disease [8]. The treatment options for hydatid cyst of the liver depend on stage, localization, size, and complications of the cysts and include nonoperative and operative methods. Operative methods include conservative and radical procedures like the classic surgical techniques and minimally invasive and laparoscopic methods. Chemotherapy with antihelmintics of benzimidazole family when used alone has limited efficacy mostly related to the accessibility of the cyst to the drug so the treatment outcome is better when used adjunctively with surgery to prevent recurrence [9]. Percutaneous drainage of liver hydatid cysts in combination with drug therapy has been found to be efficient for CE1 and CE2 types of hydatid cysts and occasionally for CE3 type but with lower success rates [10]. Radical surgical resection remains the mainstay of treatment for all patients with symptomatic disease who are good candidates for surgery [11, 12]. In conclusion, we believe that hydatid cyst can rarely reach so extremely large dimension without any additional symptom, could easily be misdiagnosed as a pseudotumoral mass, and should be considered in the differential diagnosis and in the decision for the radical therapy.
